# Establishment of transient and stable transfection systems for *Babesia ovata*

**DOI:** 10.1186/s13071-016-1439-z

**Published:** 2016-03-23

**Authors:** Hassan Hakimi, Junya Yamagishi, Yuto Kegawa, Osamu Kaneko, Shin-ichiro Kawazu, Masahito Asada

**Affiliations:** Department of Protozoology, Institute of Tropical Medicine (NEKKEN), Nagasaki University, Sakamoto 1-12-4, Nagasaki, 852-8523 Japan; Research Center for Zoonosis Control, Hokkaido University, Sapporo, 001-0020 Japan; Global Station for Zoonosis Control, GI-CoRE, Hokkaido University, Sapporo, 001-0020 Japan; Leading Program, Graduate School of Biomedical Sciences, Nagasaki University, Nagasaki, 852-8523 Japan; National Research Center for Protozoan Diseases, Obihiro University of Agriculture and Veterinary Medicine, Obihiro, Hokkaido 080-8555 Japan

**Keywords:** Bovine babesiosis, *Babesia ovata*, Stable transfection, Double cross-over homologous recombination

## Abstract

**Background:**

Bovine babesiosis is a tick-borne disease caused by several species of *Babesia* which produce acute and fatal disease in cattle and affect livestock industry worldwide. *Babesia ovata* is a benign species widespread in east Asian countries and causes anemia, particularly in cattle which are co-infected with *Theileria orientalis*. The development of genetic manipulation methods is necessary to improve our understanding of the basic biology of protozoan pathogens toward a better control of disease. Such tools have not been developed for *B. ovata*, and are the aim of this study.

**Methods:**

In this study we transfected constructs that were designed to evaluate the ability of several *B. ovata* promoter candidates to drive expression of a reporter luciferase. We found that the *elongation factor-1 alpha* intergenic region (*ef-1α* IG) and the *actin* 5’ non-coding region (NR) had highest promoter activities. To establish a stable transfection system, we generated a plasmid construct in which the *ef-1α* IG promoter drives *gfp* expression, and the *actin* 5’ NR mediates expression of the selectable marker *hdhfr*. The plasmid was designed for episomal transfection, as well as to integrate by double cross-over homologous recombination into the *ef-1α* locus. Circular or linearized plasmid was transfected by electroporation into *in vitro* cultured *B. ovata* and retention of the plasmid was facilitated by drug selection with 5 nM WR99210 initiated 48 h after transfection.

**Results:**

After one-week cultivation with WR99210, GFP-expressing parasites were observed by fluorescence microscopy. Integration of the plasmid construct into the *ef-1α* locus was confirmed by PCR, Southern blot analysis, and sequencing of recombination sites. These results confirm successful development of a stable transfection system for *B. ovata*.

**Conclusion:**

The current study provides a fundamental molecular tool to aid in molecular and cellular studies of *B. ovata*.

**Electronic supplementary material:**

The online version of this article (doi:10.1186/s13071-016-1439-z) contains supplementary material, which is available to authorized users.

## Background

Bovine babesiosis remains an economic burden in livestock industry worldwide and is caused by intraerythrocytic apicomplexan parasites of the genus *Babesia*. The most common and pathogenic species of  *Babesia* infecting cattle are *Babesia bovis*, *B. bigemina* and *B. divergens*, whose geographical distributions are defined by the prevalence of their respective tick vectors. *B. ovata* is a benign bovine *Babesia* sp. widespread in several east Asian countries [1] and causes anemia particularly in animals co-infected with *Theileria orientalis*, since both parasites are transmitted by the ixodid tick, *Haemaphysalis longicornis* [2].

Obstacles to control bovine babesiosis include the lack of effective and safe vaccines, the emergence of acaricide-resistant ticks [3], limited choices for anti-*Babesia* drugs in the field, and the emergence of drug-resistant *Babesia* strains [4]. Better understanding of the basic biology of *Babesia* spp. is crucial to design and develop new strategies for controlling the disease. In the post-genomic era in which the genome sequence information for several *Babesia* spp. is available, genetic manipulation using reverse genetic techniques can improve our understanding of the basic biology of these parasites. Transfection systems have been established for several apicomplexan parasites such as *Plasmodium falciparum* [5], *Toxoplasma gondii* [6], *Babesia bovis* [7, 8] and recently *Theileria parva* [9]. Regarding *Babesia* spp., only two reports describe stable transfection systems, for *B. bovis* [7, 8]; one used blasticidin-S/*blasticidin deaminase* and the other employed WR99210/*human dihydrofolate reductase* (*hdhfr*) as selective markers.

Several *Babesia* spp. are responsible for babesiosis in cattle causing a range of clinical symptoms from acute severe anemia and occasionally death by *B. bigemina* and *B. bovis* to a mild anemia or subclinical signs by *B. ovata*. Our group has recently derived whole genome nucleotide sequence for *B. ovata* (unpublished data). This advancement, together with the availability of genome sequences from other pathogenic bovine *Babesia* spp. [10–12], paves the way for comparative functional genomics studies and to find genes responsible for the virulence and pathogenesis of these parasites. To aid in these molecular and cellular studies, we describe herein the development of a transfection system for *B. ovata*. Since *B. ovata* infection is benign and does not cause severe clinical symptoms in cattle, stable integration of expression vectors could be used to transfer an immunogenic antigen of pathogenic *Babesia* spp. and induce protective antibody against virulent bovine *Babesia* spp. In addition, *H. longicornis*, the tick vector for *B. ovata*, is easily maintained *in vitro* and has been used to study the biology of ixodid ticks [13]. Therefore, *B. ovata* could be employed as a model organism to study the tick stage of *Babesia*, the genes responsible for tick transmission as well as parasite and tick interactions.

Since the *elongation factor-1 alpha* intergenic region (*ef-1α* IG*)* and the *actin* 5’ non-coding region (NR) contain promoter activities which conferred expression of a reporter protein in *B. bovis* [8, 14], we initially attempted to use these *B. bovis* promoters for transfection of *B. ovata*. However, these trials were not successful (unpublished data), suggesting that *B. bovis* promoters might not work in *B. ovata*. Therefore, in this study, we initially evaluated several *B. ovata* promoter candidates and then established a stable transfection system for this parasite using selected strongest promoters.

## Methods

### Parasite culture

The *B. ovata* Miyake strain was cultured *in vitro* using purified bovine red blood cells (RBCs) purchased from Nippon Bio-Test Laboratories (Tokyo, Japan) and GIT medium (Wako Pure Chemical Industries, Osaka, Japan) in a microaerophilous stationary phase system as described [15].

### Evaluation of sensitivity of *B. ovata* to WR99210

*B. ovata* was cultured in 1 ml culture medium containing 10 % bovine RBC in a 24-well plate with or without WR99210 (0, 0.5, 1, 5, 10 and 50 nM). The initial parasitemia was 0.5 % and the final parasitemia was measured on day 3. For each drug concentration, parasites were cultured in triplicate and culture medium was replaced daily. Parasitemia was calculated by examining 5000 RBCs of a prepared thin blood smear stained with Giemsa's solution.

### Plasmid constructs

**Fig. 1:**
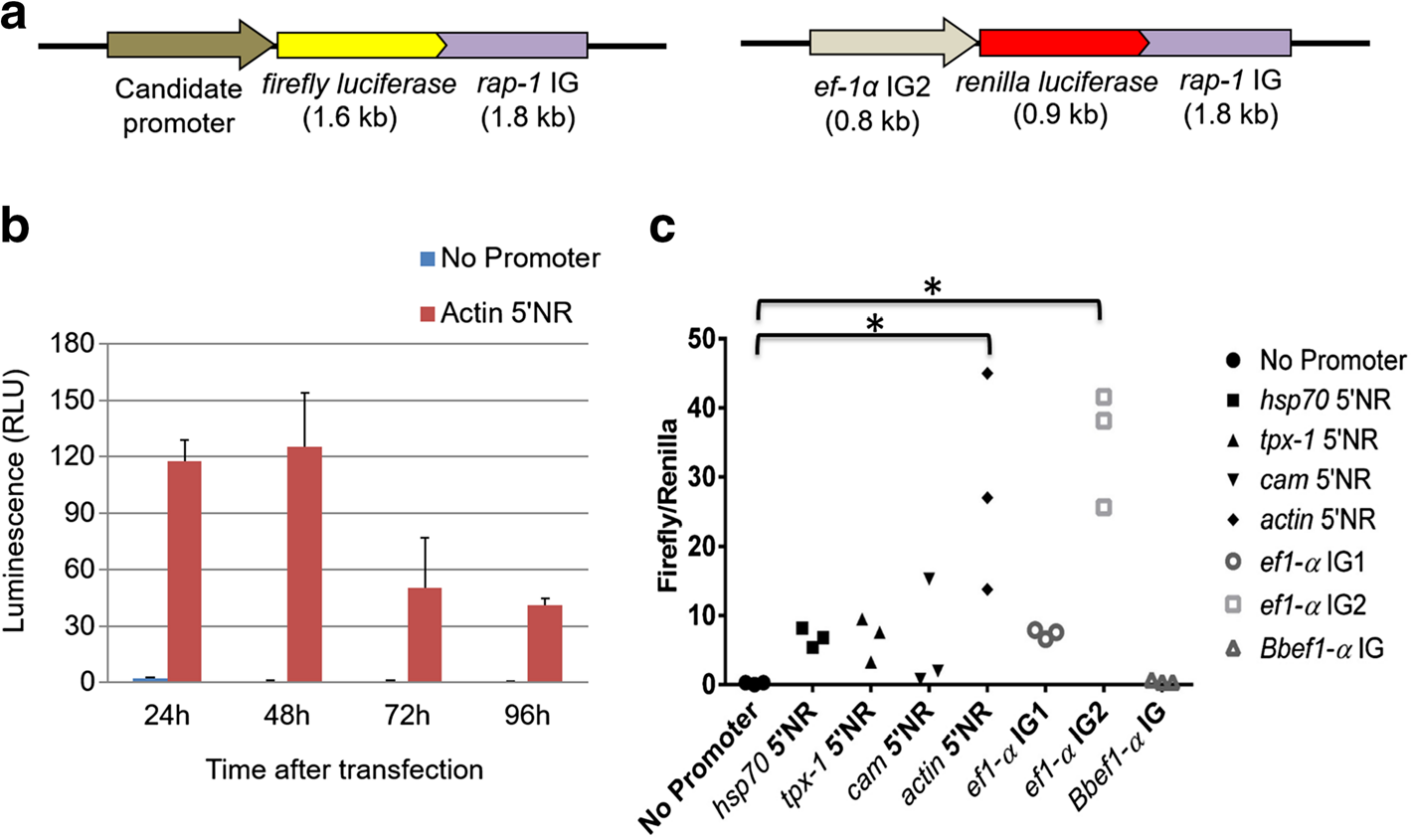
Schematic diagram of the plasmids used for transient transfection and evaluation of promoter activity. **a** Plasmid construct to evaluate the promoter activity and a Renilla luciferase-expressing plasmid for normalization. **b** Evaluation of the promoter activity of *actin* 5’NR driving luciferase expression, over a time course of 24–96 h post transfection. **c** Comparison of luciferase activity in lysates of *B. ovata* transfected with different constructs at 48 h post transfection. A promoter-less plasmid was used as a negative control. Values from 3 independent transfections are shown. Asterisks indicate statistical significance between promoter-less plasmid (No Promoter) and other promoter candidates by Dunnett’s multiple comparison test (*p* < 0.001). RLU: Relative luciferase units

For evaluation of promoter activity, the *firefly luciferase* gene was amplified by PCR from the plasmid pENT12luc using specific primers (Additional file 1: Table S1) and inserted into the EcoRV site of pBluescript plasmid vector using an In-Fusion HD Cloning Kit (Takara Bio Inc., Otsu, Japan) (Fig. 1a). The *B. ovata rhoptry associated protein-1* intergenic region (*rap-1* IG) was amplified by PCR from *B. ovata* genomic DNA using specific primers and inserted into the BamHI site of pBluescript. The *B. ovata ef-1α* IG, 5’ NR of *actin*, *heat shock protein 70* (*hsp70*), *calmodulin* (*cam*), *thioredoxin peroxidase 1*(*tpx-1*) and *B. bovis ef-1α* IG were amplified by PCR and cloned into the HindIII site of pBluescript to be evaluated for their promoter activities. To construct an internal control plasmid to normalize the promoter activity, a DNA fragment encoding *Renilla reniformis* luciferase was amplified from pHRH (a gift from Dr. Kirk Deitsch, Weill Cornell Medical College, USA) using specific primers (Additional file 1: Table S1) and inserted into the EcoRV site of pBluescript plasmid vector (Fig. 1a). Renilla luciferase was driven by *ef-1α* IG2 and *rap-1* IG used as terminator. The sequences from all novel candidate promoters were deposited in DDBJ/EMBL/GenBank nucleotide database with the accession numbers LC101765-69.

**Fig. 2:**
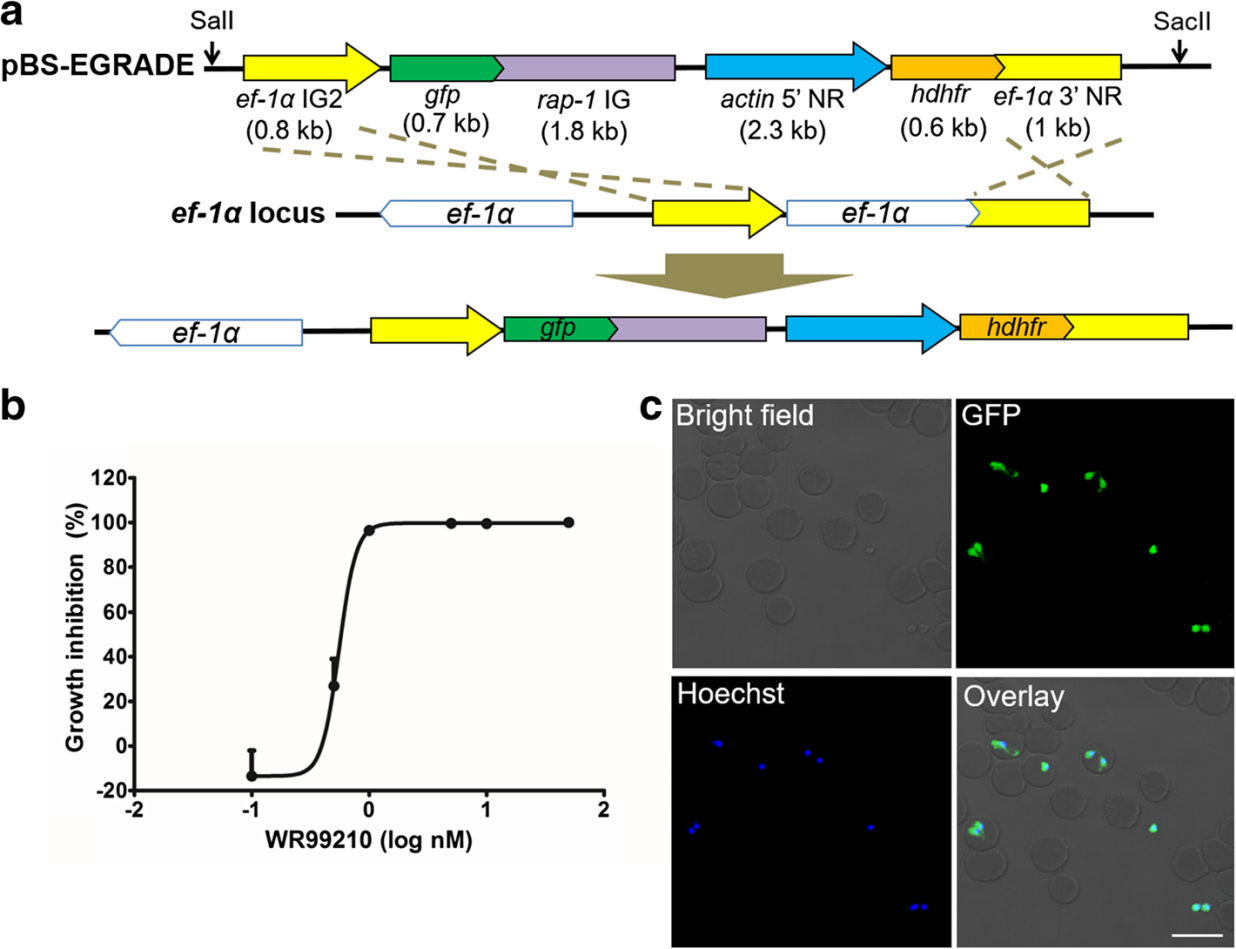
Schematic diagram of GFP-expressing plasmid construct, growth inhibition of *B. ovata* by WR99210 and fluorescence microscopy images of stably expressing GFP *B. ovata*. **a** Plasmid construct for stable GFP expression (pBS-EGRADE) showing the recombination sites for integration into the *ef-1α* locus by homologous double cross-over recombination. The restriction sites for linearization (SalI and SacII) are shown. **b** Growth inhibition rate of *B. ovata* in the absence or presence of different concentrations of WR99210. All data are expressed as mean + SEM of triplicate culture. **c** Live fluorescence microscopy images of GFP-expressing parasites. The pictures are taken from the pBS-EGRADE genome integrated isolate 1. The parasite nucleus was stained with Hoechst33342. *Scale-bar*: 10 μm

A schematic diagram of GFP-expressing plasmid (pBS-EGRADE) is shown in Fig. 2a. The plasmid was designed for episomal transfection as well as integration into the *ef-1α* locus of *B. ovata*. The drug selection and reporter gene cassettes were separated in order to drive *hdhfr* and *gfp* with different promoters. All the PCR primers used for plasmid construction are listed in Table 1 and the restriction sites are underlined. To construct the reporter cassette, *gfp* was amplified from a *B. bovis* GFP expressing plasmid [8] and cloned into the HindIII site of pBluescript using an In-Fusion HD Cloning Kit. To produce a strongly expressing GFP parasite, the *ef-1α* IG2 which showed strong promoter activity by luciferase assay was cloned into a ClaI site at the 5’ flanking region of *gfp. B. ovata rap-1* IG was cloned into an EcoRV site at the 3’ flanking region of *gfp*. For construction of the drug selection cassette, *hdhfr* was amplified from a *B. bovis* GFP-expressing plasmid [8] and cloned into the BamHI site of pBluescript. The *hdhfr* gene was flanked by *actin* 5’ NR and *ef-1α* 3’ NR at the 5’ and 3’ flanking regions, respectively. The resulting *hdhfr* expression cassette was amplified with a primer pair, BoADE-F-EcoRI-IF and BoADE-R-EcoRI-IF (Table 1), and cloned into the EcoRI site of the reporter cassette to produce the pBS-EGRADE plasmid. All plasmids were purified using a Qiagen Plasmid Maxi kit (Qiagen, MD, USA) following the manufacturer’s instructions and the inserted DNA sequences were confirmed by sequencing. The sequence of pBS-EGRADE was deposited into DDBJ/EMBL/GenBank nucleotide database with the accession number LC101772.

**Table 1:** List of primers used to construct GFP-expressing plasmid, pBS-EGRADE

Primer	Sequence
BoefIG2-F-ClaI-IF	AGGTCGACGGTATCGATCACTCATTTAGATTGCGA
BoefIG2-R-ClaI-IF	ATATCAAGCTTATCGATCTTGTTTAAGGTTTAACGA
eGFP-F-HindIII-IF	CAAGATCGATAAGCTTATGGTGAGCAAGGGCGAGGA
eGFP-R-HindIII-IF	TCCCGATATCAAGCTTTTACTTGTACAGCTCGTCCA
BoRAPIG-F-EcoRV-IF	ATCGATAAGCTTGATATCGGGAGCAGAGCTCTGGCCA
BoRAPIG-R-EcoRV-IF	CTGCAGGAATTCGATATCCGTAAAGCGAGACGGGGA
BoActin5UTR-F-SmaI-IF	GAATTCCTGCAGCCCGGGCACATAAAGGTACCTATGCT
BoActin5UTR-R-SmaI-IF	ACTAGTGGATCCCCCGGGCTTGGCGAAATTTTACTCCT
hDHFR-F-BamHI-IF	CAAGCCCGGGGGATCCATGGTTGGTTCGCTAAACT
hDHFR-R-BamHI-IF	ACGTACTAGTGGATCCTTAATCATTCTTCTCATATAC
Boef3UTR-F-SpeI-IF	CGGGGGATCCACTAGTACGTTGCGCCTTTAGGTGCA
Boef3UTR-R-SpeI-IF	CCGCTCTAGAACTAGTGGTGTGAGCATTGCTTGTC
BoADE-F-EcoRI-IF	TACGGATATCGAATTCCTGCAGCCCGGGCACATAAAGG
BoADE-R-EcoRI-IF	CGGGCTGCAGGAATTCACTAGTGGTGTGAGCATTGC

Restriction enzyme sites are underlined

### Transfection of parasites

To prepare *B. ovata*-infected RBCs (iRBCs) for transfection, cell pellets were washed twice with PBS and one time with cytomix buffer (120 mM KCl, 0.15 mM CaCl_2_, 10 mM K_2_HPO_4_, 10 mM KH_2_PO_4_, 25 mM HEPES, pH 7.6, 2 mM EGTA, 5 mM MgCl_2_, 100 μg/ml bovine serum albumin, and 1 mM hypoxanthine). One hundred microgram of the circular plasmid in 50 μl of cytomix was mixed with 50 μl of Amaxa nucleofector human T-cell solution, and then combined with 100 μl of washed iRBCs. To promote genome integration of the plasmid in order to produce *B. ovata* stably expressing GFP, the pBS-EGRADE plasmid was linearized by incubating overnight with SalI and SacII. The resulting linear plasmid would favor integration into the *ef-1α* locus of *B. ovata*. Parasite-iRBCs were transfected with the circular or linearized plasmids by electroporation using a nucleofector device with a program v-024 (Amaxa Biosystems, Cologne, Germany) [8] and were immediately transferred to 1 ml culture containing 10 % of bovine RBCs. To select GFP-expressing transgenic parasites, WR99210 was added two days after the transfection. The parasite population transfected with the linearized plasmid was cloned by limiting dilution.

### Luciferase assay

A luciferase assay was performed to evaluate activities of candidate promoters. Transient transfections were conducted by introducing 50 μg of each promoter plasmid expressing firefly luciferase together with 50 μg of renilla luciferase-expressing plasmid into *B. ovata*-iRBCs. The luciferase assay was performed 48 h after transfection. A parasite pellet was prepared for the luciferase assay as described [16] and resuspended in 230 μl of freshly-prepared Promega’s 1X cell culture lysis reagent (Promega, Madison, WI, USA). The pellet was incubated for 15 min at room temperature (RT) for complete lysis and briefly centrifuged to remove the cell debris. Seventy-five microliters of cell lysate were mixed with 75 μl of the Dual-Glo luciferase substrate. The mixture was kept for 10 min at RT and the luminescence was measured for 10 s using a microplate reader (Wallac 1420; PerkinElmer, Turku, Finland). Immediately after measurement of firefly luciferase activity, 75 μl of Dual-Glo Stop & Glo reagent was added to each well of the plate. Renilla luciferase activity was measured after 10 min incubation at RT. Readings from mock transfected parasites were subtracted from the firefly and renilla luciferase readings and the resulting values of firefly luciferase activity were normalized using the renilla luciferase activity for each sample. To evaluate promoter activities, three independent transfections were done for each promoter and each luciferase assay was performed in triplicate.

### Biostatistical analysis of promoter activities

The normalized luciferase activities were plotted using GraphPad Prism6. Activity of each promoter candidates was examined by Dunnett’s multiple comparison test with a promoter-less (control) plasmid. The promoter activity was considered to be significantly different from the control if *P-*value was below 0.05.

### PCR to confirm the integration of pBS-EGRADE into *ef-1α* locus

Three sets of primers were designed to confirm the integration of pBS-EGRADE into the *ef-1α* locus by specific amplification of DNA fragments surrounding the 5’ recombination site, the 3’ recombination site and the *ef-1α* locus (Additional file 1: Table S1). To examine the 5’ recombination event of the plasmid construct, a primer pair Boef1α-integ-F and eGFP-R-HindIII-IF was used to amplify a 2.2 kb DNA fragment. To confirm the 3’ recombination event of the plasmid construct, a primer pair hDHFR-F-BamHI-IF and Boef1α-integ-R was used to amplify a 1.8 kb DNA fragment. To examine if the *ef-1α* gene was replaced with the plasmid construct, a primer pair Boef1α-F2 and Boef1α-integ-R was used, by which a 7.7 kb DNA fragment would be amplified for the pBS-EGRADE-integrated parasites or a 3.6 kb DNA fragment for the wild type and episomally transfected parasites. The nucleotide sequences of the amplified DNA fragments containing the 5’ or 3’ recombination sites were confirmed by sequencing.

### Southern blot analysis

Genomic DNA was extracted from wild type and 2 parasite isolates transfected with linearized pBS-EGRADE. Five micrograms of DNA were digested overnight with 100 units of NotI and PacI, separated by agarose gel electrophoresis and transferred onto HyBond N+ (GE Healthcare, Buckinghamshire, UK). Two probes were used, one corresponding to the complete open reading frame (ORF) of *gfp* and the other 1 kb length of *ef-1α* 3’NR, and were labeled and hybridized with the AlkPhos Direct kit (GE Healthcare) according to the manufacturer’s instructions. The signal was developed with CDP-star detection reagent (GE Healthcare) and detected with a multipurpose CCD camera system (LAS-4000 mini EPUV; Fujifilm, Japan).

## Results

### Evaluation of the *B. ovata* promoter activities

In order to evaluate promoter activity and to select promoters to be used for stable transfections, candidate promoters were chosen which were previously used for *B. bovis* or *Plasmodium*. The evaluated candidate promoters were (i) e*f-1α* IG, which has a bidirectional promoter activity in *B. bovis* [17]; (ii) *actin* 5’NR, which showed strong promoter activity in *B. bovis* [8]; (iii) *tpx-1* 5’NR, which was shown to constitutively express in *Plasmodium* spp. [18, 19]; and (iv) *cam* 5’NR and *hsp70* 5’NR, which were used for *P. falciparum* [5, 20]. Similar to the *B. bovis ef-1α* gene locus [17], two *ef-1α* gene copies are oriented head to head in the *B. ovata* genome, and thus the IG region was expected to have a bidirectional promoter activity. To independently evaluate the two promoter activities in the *ef-1α* IG region, two plasmids termed IG1 and IG2 were constructed (Fig. 1c). Our initial analysis of *rap-1* region in *B. ovata* revealed at least three copies of *rap-1a* and *rap-1b* in the genome. This architecture is similar to what was observed for the *B. bigemina* genome in which five copies of *rap-1a* and *rap-1b* are tandemly arranged and an additional copy of *rap-1b* and a copy of *rap-1c* are located at 3’ of these gene loci [21]. The intergenic region between *rap-1a* and *rap-1b* which contains the 3’ NR of *rap-1a* was selected as a terminator.

In the initial trials for transient transfection, we introduced 50 μg of plasmid constructs with transfection conditions of low voltage/high capacitance (0.31 kV, 1 mF) or high voltage/low capacitance (1.25 kV, 25 μF) using a Gene Pulser Xcell apparatus (Bio-Rad, Richmond, CA) [14]. Parasite lysates did not show any luciferase activity indicating the failure in introducing plasmid into the parasite using these conditions, possibly due to harmful effects of electroporation to the parasites. Because *P. falciparum* is able to uptake plasmid DNA and efficiently express exogenous proteins following infection of erythrocytes which are preloaded by electroporation into the RBC cytosol [22], we preloaded 50 μg of plasmid DNA to the bovine RBCs for the transfection experiment. Again, however, parasite lysates did not show any luciferase activity after this procedure. The erythrocytic cycle of *B. ovata* (7–10 h) [23] is shorter than that of *P. falciparum* (48 h), which might be the reason for the failure of the transfection with plasmid-preloaded RBC. In a third attempt nucleofection using an Amaxa nucleofector device with 50 μg of plasmid DNA was successful for transient expression experiments (data not shown). Increasing plasmid DNA to 100 μg showed more efficient transformation and was used for the evaluation of promoter activity and stable transfection.

We first determined the best time for evaluation of the promoter activity for *B. ovata* using a plasmid expressing luciferase driven by the *B. ovata actin* 5’NR. Parasite lysates were prepared at 24, 48, 72 and 96 h post transfection for the luciferase assay. We found that while parasites transfected with a promoter-less construct (negative control) did not show luciferase activity, the peak of luciferase activity for *actin* 5’NR was at 48 h post transfection; thus we selected this time point to evaluate luciferase activities (Fig. 1b). Simultaneous expression of firefly luciferase under different promoters and renilla luciferase under the identical promoter in *Plasmodium* spp. enables to normalize the firefly luciferase value using renilla luciferase value as a transfection efficiency control [20, 24]. Therefore, firefly luciferase luminescence values were normalized with the renilla luciferase values from the same samples. By three independent transfection experiments, *ef-1α* IG2 and *actin* 5’NR showed significantly higher values than promoter-less (control) plasmid (*P* < 0.001), whereas the luciferase values from *hsp70* 5’NR, *tpx-1* 5’NR, *cam* 5’NR and *ef-1α* IG1 were not significantly different when compared to the control (*P* > 0.05) (Fig. 1c). The *B. bovis ef-1α* IG construct, for which a promoter activity was validated in *B. bovis* [8], was not able to express *firefly luciferase* in *B. ovata*, indicating that *B. bovis ef-1α* IG was not recognized by the *B. ovata* transcriptional or translational machinery.

### Growth of *B. ovata* is inhibited by WR99210 at nanomolar concentration

WR99210 has been successfully used to select for stable transfection of *B. bovis* [8]. To examine whether WR99210 could inhibit the growth of *B. ovata* at low concentration, and to determine a suitable concentration to select the transgenic parasites, *B. ovata* was cultured in the presence of different concentrations of WR99210 from 0 to 50 nM. The experiment was done in triplicate wells and parasitemia was calculated on day 3 after adding the drug into the culture (Fig. 2b). The calculated IC_50_ was 0.56 ± 0.01 nM and 5 nM WR99210 completely inhibited the growth of *B. ovata*; thus we employed 5 nM thereafter for the selection of stable GFP-expressing parasites.

### Establishment of the *B. ovata* line stably expressing GFP

**Fig. 3:**
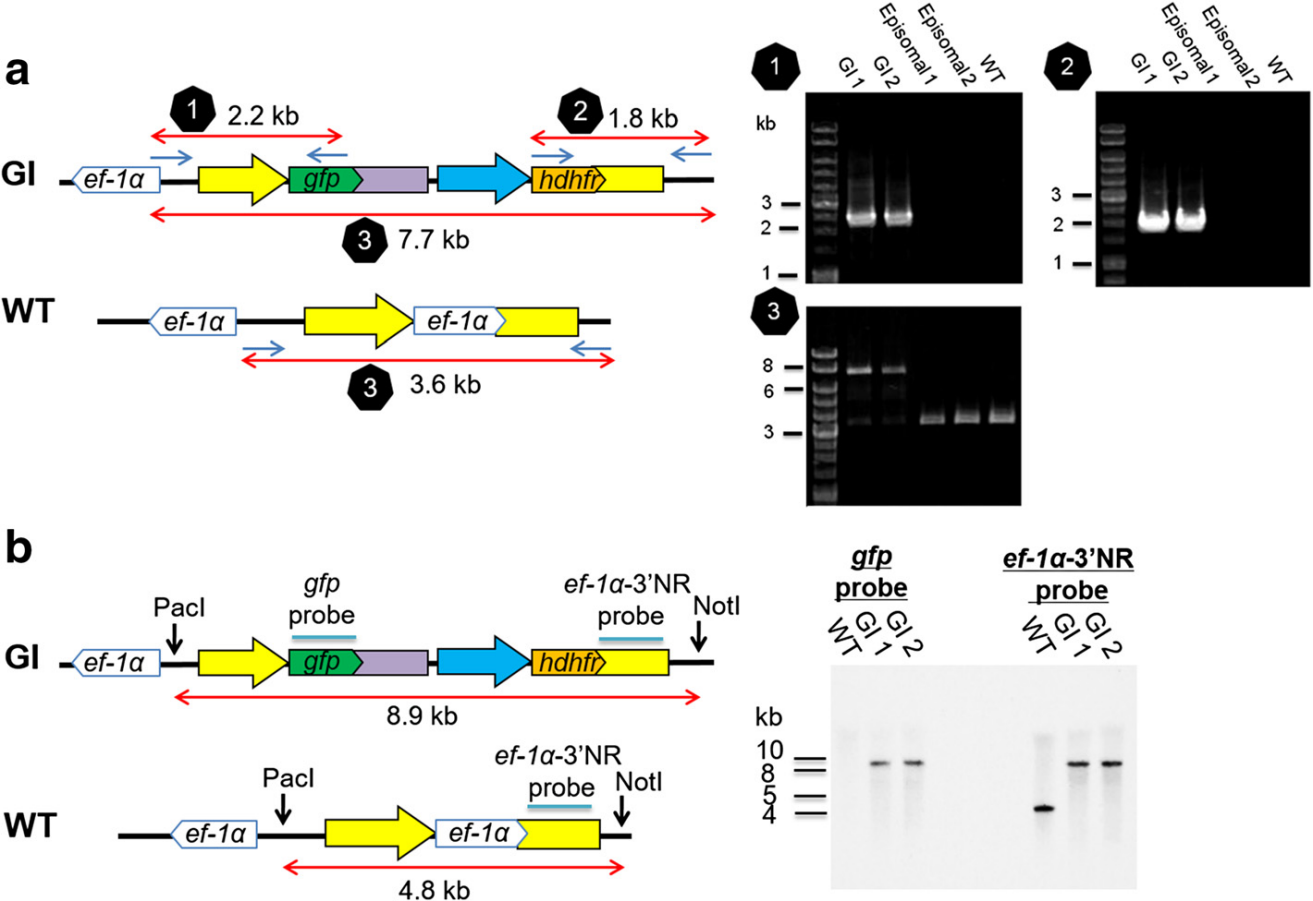
Confirmation of the integration of pBS-EGRADE into the *ef-1α* locus. **a** Schematic diagram and PCRs to confirm the integration of pBS-EGRADE into *ef-1α* locus. PCR-1, −2 and −3 are done with primer sets Boef1α-integ-F/eGFP-R-HindIII-IF, hDHFR-F-BamHI-IF/Boef1α-integ-R and Boef1α-F2/Boef1α-integ-R, respectively. **b** Schematic diagram and Southern blot analysis to confirm the integration of pBS-EGRADE into *ef-1α* locus. Five microgram of samples genomic DNA were digested with PacI and NotI, and hybridized with *gfp* and *ef-1α* 3’NR probes. GI: genome integrated; Episomal: two independent transfectants with circular plasmids; WT: wild type

**Fig. 4:**
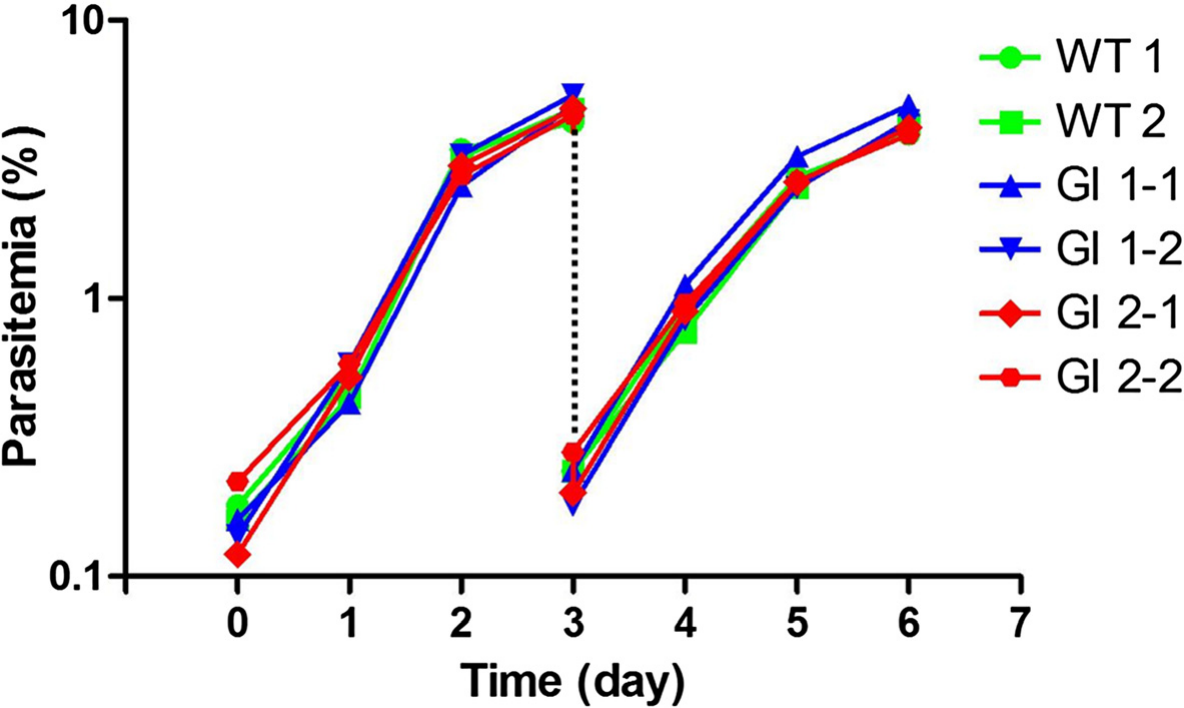
Growth curve of different lines of *B. ovata*. Wild type (WT) and genome integrants (GI) were cultured continuously by sub-culturing every 3 days and parasitemia monitored daily

Approximately one week after addition of WR99210, GFP-expressing parasites appeared in cultures which were transfected with either circular or linearized plasmids (Fig. 2c). The integration of pBS-EGRADE into the *ef-1α* locus was initially evaluated by PCR. The diagnostic PCR-1 and −2 primer pairs successfully amplified 2.2 and 1.8 kb DNA fragments, respectively, using DNA template from genome integrated (GI) isolates; and the amplified DNA fragments were validated by sequencing (Fig. 3a). These DNA fragments sequences were deposited into DDBJ/EMBL/GenBank nucleotide database with the accession numbers LC101770 and LC101771. No amplified products were detected using DNA template from episomal transfectants or wild type parasites. In addition, PCR-3 primer pair amplified a 7.7 kb DNA fragment, a size expected after the insertion of the pBS-EGRADE construct into the *ef-1α* locus. In contrast, a 3.6 kb DNA fragment was amplified for wild type and episomal transfectants, indicating that their *ef-1α* locus was intact. Overall, the PCR results confirmed the integration of the plasmid construct into the *ef-1α* locus in the GI1 and GI2 parasite lines. We further confirmed the structure of the integration site by Southern blot analysis (Fig. 3b). The *gfp* and *ef-1α* 3’NR probes both detected a single 8.9 kb band for GI isolates 1 and 2, while only the *ef-1α* 3’NR probe detected a single 4.8 kb band and the *gfp* probe did not detect any band for wild type parasites. These results confirmed the correct integration of pBS-EGRADE into the *ef-1α* locus. In addition, the growth of GI isolates was comparable with wild type parasites, indicating that disruption of one copy of the *ef-1α* gene did not affect the growth of parasites in the *in vitro* culture condition (Fig. 4).

## Discussion

In this study we transiently introduced plasmid DNA into *B. ovata* in order to evaluate promoter activity. With this transient expression we evaluated the strength of promoter activity for a panel of *B. ovata* promoters, as well as for the *B. bovis ef-1α* IG promoter region. Evaluation of a range of promoter types and activities increases the options of promoters to be chosen to express transgenes in accordance the purposes of a given study. Herein, we aimed to establish a transfection system using GFP as a reporter protein, and therefore the *ef-1α* IG2 was chosen to drive *gfp* and the *actin* 5’NR to drive *hdhfr* based upon their relatively strong promoter activities. Unlike *Plasmodium* spp., for which *ef-1α* promoter region from one species works in other species, the *B. bovis ef-1α* IG promoter region was not able to express luciferase in *B. ovata*, and this indicates that *B. bovis ef-1α* IG was not recognized by *B. ovata*’s transcription or translation system.

The first report of stable transfection of *B. bovis* targeted the *ef-1α* locus for genome integration of a plasmid construct [7]. Preliminary investigation of the *ef-1α* locus sequence in *B. ovata* showed that two identical genes are located head to head and are separated by a 1.4 kb IG region (unpublished data), similar to the pattern reported for *B. bovis* and *Plasmodium* [17, 25]. Replacing one of two *ef-1α* in *Plasmodium* or *B. bovis* by homologous recombination did not significantly affect the growth of the parasites [7, 26, 27] and thus we considered that one of the *ef-1α* gene loci could be targeted as an integration site of exogenous genes into the genome. As expected, we were able to use this gene locus for the genome integration in *B. ovata*, and destruction of one of the *ef-1α* gene loci did not affect the growth of GI isolates, suggesting the feasibility of this locus as a site for the introduction of foreign genes.

For the selection of GFP-expressing parasites we employed *hdhfr* as a selectable marker which confers resistance to WR99210 and pyrimethamine [28]. It was reported that in most cases the selection of *B. bovis* using *hdhfr*/pyrimethamine resulted in the selection of naturally resistant parasites, which challenged the use of this selection system for *B. bovis* [14]. However, the *hdhfr*/WR99210 system was successfully employed for *B. bovis* in our former study, in which natural resistant parasites were not selected [8]. Thus we used WR99210 instead of pyrimethamine. The IC_50_ of WR99210 against *B. ovata* was 0.56 ± 0.01 nM, which is comparable to that of *B. bovis* (1 nM) [8]. The transfectants selected with *hdhfr*/WR99210 in this study emerged as early as 7 days after adding the drug and continued expressing GFP for more than 7 months after transfection (data not shown), indicating the feasibility of using this selection system for *B. ovata*.

In a recent report two selection systems, blasticidin-S/*blasticidin deaminase* and WR99210/*hdhfr*, were successfully used sequentially in a double transfection system for complementation of *tpx-1* gene knockout and subsequent recovery of the phenotype for *B. bovis* [29]. This method could be used not only for gene knockout complementation studies but also for the development of double gene knockouts to investigate biological aspects of this parasite, such as invasion and egress, where multiple genes may be involved. In addition, the available gene knockout technologies for *T. gondii* and *P. falciparum* were further advanced by the CRISPR/Cas9-based genome editing strategy [30, 31]. The CRISPR/Cas9 system has also been successfully employed for genetic modification of the diarrheal protozoan parasite, *Cryptosporidium parvum* [32], indicating the feasibility of this genome editing system in various organisms. These developments together with conditional systems for regulation of gene expression at the genomic, transcriptional or protein level could be applied for *Babesia* spp. to investigate genes responsible for survival, virulence, transmission and immunity of bovine babesiosis.

## Conclusion

Here, we established a transient and stable transfection systems for *B. ovata* and successfully inserted a foreign gene *via* integration and disruption of one copy of the *ef-1α* gene. This advancement in methodology enables us to investigate gene functions in *B. ovata* by targeted gene disruption, complementation and tagging of target proteins.

## Additional file

Additional file 1: Table S1. List of primers used for promoter evaluation constructs and PCRs to confirm the integration of pBS-EGRADE into *ef-1α* locus. (PDF 162 kb)

## Abbreviations

*ef-1α*: *Elongation factor-1 alpha*
IG: Intergenic region
*rap-1*: *Rhoptry associated protein-1*
*hdhfr*: *Human dihydrofolate reductase*
RBC: Red blood cell
*hsp70*: *Heat shock protein 70*
*tpx-1*: *Thioredoxin peroxidase-1*
*cam*: *Calmodulin*
iRBC: Infected RBC
RT: Room temperature
GI: Genome integrated
ORF: Open reading frame
