# Supplementary material for: Establishment of transient and stable transfection systems for Babesia ovata
Source: Parasit Vectors. 2016 Mar 23;9:171. doi: 10.1186/s13071-016-1439-z (PMC4806448; doi:10.1186/s13071-016-1439-z)
Supplement: Additional file 1: Table S1. — List of primers used for promoter evaluation constructs and PCRs to confirm the integration of pBS-EGRADE into ef-1α locus. (PDF 162 kb) [file 13071_2016_1439_MOESM1_ESM.pdf]

Hakimi et al. Supplementary Table S1

List of primers used for promoter evaluation constructs and PCRs to confirm the integration of pBS-EGRADE into *ef-1α* locus

Primers	Sequence
Luc-F-ECORV-IF	GCCAAGAAGCTTGATATCATGGAAGACGCCAAAAACAT
Luc-R-ECORV-IF	CTGCAGGAATTCGATATCTTACAATTTGGACTTTCCGCC
Renilla-F-ECORV-IF	AACAAGAAGCTTGATATCATGACTTCGAAAGTTTATGATCCA
Renilla-R-ECORV-IF	CTGCAGGAATTCGATATCTTATTGTTCATTTTTGAGAAC
Boef1IG1-F-HindIII-IF	CGGTATCGATAAGCTTCTTGTTTAAGGTTTAACGAT
Boef1IG1-R-hindIII-IF	CCATGATATCAAGCTTGTGTATGAACGTTCTGTGC
Boef1IG2-F-HindIII-IF	CGGTATCGATAAGCTTCACTCATTTAGATTGCGACAT
Boef1IG2-R-hindIII-IF	CCATGATATCAAGCTTCTTGTTTAAGGTTTAACGAT
Bohsp5UTR-F-HindIII-IF	CGGTATCGATAAGCTTCGTTGGCGTGTCTAAAGAAG
Bohsp5UTR-R-hindIII-IF	CCATGATATCAAGCTTTTTCGGATGCTTGTTCTACAGTC
BoCal5UTR-F-HindIII-IF	CGGTATCGATAAGCTTCACGGCACGTCATCACTCAT
BoCal5UTR-R-hindIII-IF	CCATGATATCAAGCTTCGTGTTATCCTTGTTATCGCC
BoTPx5UTR-F-HindIII-IF	CGGTATCGATAAGCTTAACGTTTCGAGAATTTACTTC
BoTPx5UTR-R-hindIII-IF	CCATGATATCAAGCTTTTTCAATGTATAGGGAATAATAAG
BoAct5-F-Hind III-IF	CGGTATCGATAAGCTTCACATAAAGGTACCTATGCTTCG
BoAct5-R-Hind III-IF	GCATGATATCAAGCTTCTTGGCGAAATTTTACTCCTGC
BoRAP3-F-BamH I-IF	GCAGCCCGGGGGATCCGGGAGCAGAGCTCTGGCCATAG
BoRAP3-R-BamH I-IF	TAGAACTAGTGGATCCCGTAAAGCGAGACGGGGACATTGC
Boef1α-integ-F	GTAGATCAGGTGACCAGTGGTG
Boef1α-integ-R	GTGATCGTCCACGGCACATGC
Boef1α-F2	GACACGTCATATGGCAGTTGTG

Restriction enzyme sites are underlined.
